# From Healthy Eating to Positive Mental Health in Adolescents: A Moderated Mediation Model Involving Stress Management and Peer Support

**DOI:** 10.3390/nu17203305

**Published:** 2025-10-21

**Authors:** Inmaculada C. Rodríguez-Rojo, Montserrat García-Sastre, Cecilia Peñacoba-Puente, Daniel Cuesta-Lozano, Leonor García-Rodríguez, Patricia Blázquez-González, Patricia González-Alegre, Juan Manuel López-Reina-Roldán, Raquel Luengo-González

**Affiliations:** 1 Community Care and Social Determinants of Health (CUYDET), Nursing and Physiotherapy Department, Universidad de Alcalá, 28805 Alcalá de Henares, Spain; inmaculada.rr@uah.es (I.C.R.-R.); mmontserrat.garcia@uah.es (M.G.-S.); raquel.luengo@uah.es (R.L.-G.); 2 Center for Cognitive and Computational Neuroscience (C3N), Universidad Complutense, 28040 Madrid, Spain; 3 Psychology Department, Universidad Rey Juan Carlos, 28933 Madrid, Spain; 4 Nursing Department, University San Pablo-CEU, CEU Universities, 28660 Boadilla del Monte, Spain; leonor.garciarodriguez@ceu.es; 5 Department of Nursing, Red Cross Nursing School, 28003 Madrid, Spain; 6 Center of Mental Health, University Hospital Principe de Asturias, 28805 Alcalá de Henares, Spain; patricia.gonzaleza@uah.es; 7 Group for Research in Nursing Care, Gregorio Marañón, Health Research Institute (IiSGM), 28009 Madrid, Spain

**Keywords:** adolescence, nutrition, stress management, peer support, positive mental health, mediation effects, moderated mediation effects

## Abstract

**Background:** Adolescent mental health is a growing public health concern, with increasing prevalence of anxiety, depression, and emotional dysregulation. While nutrition is a recognized factor in physical health, its role in mental well-being, especially through holistic models, remains underexplored. **Objectives:** This study examines a moderated mediation model in which perceived healthy eating (self-rated diet quality) was associated with positive mental health (PMH) in adolescents, mediated by stress management and moderated by peer social support. **Methods:** A cross-sectional study was conducted with a sample of 505 adolescents aged 13 to 15 years (mean age 13.62). Using PROCESS Model 14, we tested a moderated mediation model where perceived nutrition served as the predictor, stress management as the mediator, and PMH as the outcome. Peer support was included as a moderator of the mediation pathway. Gender, age, nationality and educational variables were controlled for in the analysis. **Results:** The model explained 36% of the variance in PMH. Perceived healthy eating was significantly associated with better stress management (B = 0.20, *p* < 0.001), which in turn was related to higher levels of PMH (B = 6.38, *p* < 0.001). Peer support played a moderating role between stress management and PMH. **Conclusions:** These findings underscore the importance of promoting nutritional awareness and adolescents’ self-perception of healthy eating to support both physical and emotional well-being. Interventions should adopt a holistic approach that integrates emotional regulation strategies and leverages peer influence to enhance mental health outcomes. Given the alarming rates of mental health issues in youth, nutrition-based programs that incorporate psychosocial components may offer a promising avenue for prevention and promotion.

## 1. Introduction

Adolescence is a distinct and dynamic stage of development marked by deep biological, emotional, and social transformations. During this period, hormonal changes and ongoing brain maturation in regions involved in emotional regulation and social cognition interact with heightened sensitivity to social feedback, creating both opportunities for growth and increased vulnerability. Many psychiatric conditions, including depression, anxiety, and eating disorders, first emerge in adolescence, often shaped by how young people manage stress and navigate their evolving social environments [1].

According to the World Health Organization (WHO), approximately one in seven adolescents aged 10–19 experience a mental health disorder, accounting for 15% of the global disease burden in this age group [2]. In Spain, recent data from the 2023–2024 UNICEF Spain and University of Seville Youth Opinion Barometer reveal that 41% of adolescents reported experiencing or believing they had experienced a mental health problem in the past year. However, over one-third did not speak to anyone about it, and more than half did not seek help, underscoring the persistent stigma surrounding adolescent mental health [3]. Interestingly, adolescents also identified a number of internal and external factors that support their well-being, including healthy sleep (74.9%), physical activity (62.3%), and balanced nutrition (50.6%) as well as strong family relationships (82.6%) and peer support (82%).

Within this context, there is growing recognition of the role that lifestyle factors, particularly nutrition, physical activity, and sleep, play in both the prevention and treatment of mental health disorders in youth. These factors appear to influence mental health through neurobiological and psychosocial pathways, highlighting the importance of assessing and optimizing these lifestyle factors in clinical practice [4]. Despite increasing awareness of their benefits, many adolescents fail to meet lifestyle recommendations, often due to emotional distress, low motivation, or limited support, highlighting the need for accessible and integrated interventions [5]. Evidence from school-based and youth-focused mental health programs supports the inclusion of psychoeducation, body image, stress management, and health-promoting behaviors as key components of adolescent well-being [6].

Among lifestyle factors, nutrition has gained special attention. Healthy dietary patterns and specific nutrients, such as omega-3 fatty acids, vitamin D, and increased consumption of fruits and vegetables, have been consistently linked to reduced symptoms of depression and anxiety, improved emotional functioning and greater resilience [7,8]. In contrast, poor diet quality (i.e., those high in processed food and sugary beverages) is associated with greater psychological distress, specifically, depression, and reduced well-being [9,10,11]. Other systematic reviews have already observed these associations, while also calling for more rigorous methodological approaches that address confounding factors such as socioeconomic status [12]. Adolescents with greater cooking abilities also tend to report better nutritional habits, higher emotional well-being, and stronger family connections, suggesting that food-related behaviors may serve as both psychological and relational tools [13].

Despite increasing evidence linking nutrition and mental health, the mechanisms underlying this relationship remain complex and not fully understood. One plausible psychological pathway is stress regulation, particularly adolescents’ perceived ability to manage stress in daily life. Bremner et al. [14] emphasize the central role of stress in the bidirectional relationships between diet, mood, and mental disorders, suggesting that stress may act both as a consequence and as a contributing factor in the diet-mental health interaction. Complementing this, Zhao et al. [15] found that the relationship between healthy dietary habits and fewer emotional and behavioral problems in children was significantly mediated by self-concept, a psychological resource closely linked to emotional regulation and perceived control. Similarly, Mancone et al. [16] demonstrated that nutrition education interventions promoting food literacy and self-regulation skills led to improvements in adolescents’ eating behaviors and overall health outcomes. Therefore, adolescents who perceive improvements in their dietary habits may also experience greater emotional self-regulation and a sense of control, which enhances their ability to cope with stress [16].

In addition to internal coping mechanisms, the social environment plays a central role in adolescent mental well-being. Supportive interpersonal context, especially high-quality relationships with peers and family, are not only protective against psychological distress but also essential for the adolescents’ healthy development of identity and self-esteem [17,18,19]. Adolescents who feel supported by their peers tend to experience lower anxiety and depression, improved emotional regulation, and stronger resilience [20,21,22,23]. Peer relationships also enhance a sense of belonging and connection, which may potentiate the benefits of other health-promoting behaviors. Furthermore, peers are considered to play an important role in adolescents’ eating habits. In fact, support from both parents and peers was associated with healthier eating habits in adolescents [24].

Under this scenario, positive mental health (PMH) offers a valuable framework for assessing adolescent well-being from models of positive psychology. Rooted in the salutogenic model, PMH emphasizes the presence of personal and social resources that enable individuals to manage adversity and thrive [25,26,27,28]. The PMH approach considers that a mentally healthy person is not only free of clinical symptoms, but also displays psychological resilience, adaptability, and healthy social relationships.

Furthermore, this concept has generated considerable interest. Studies have shown that PMH is linked to greater life satisfaction, academic success, and long-term well-being in adolescents [29,30]. As noted, various studies link nutrition with mental health, but most do so from the perspective of illness [8,9,10,11]. To our knowledge, no previous studies have suggested the perception of healthy eating as a predictor of PMH in particular.

**Figure 1:**
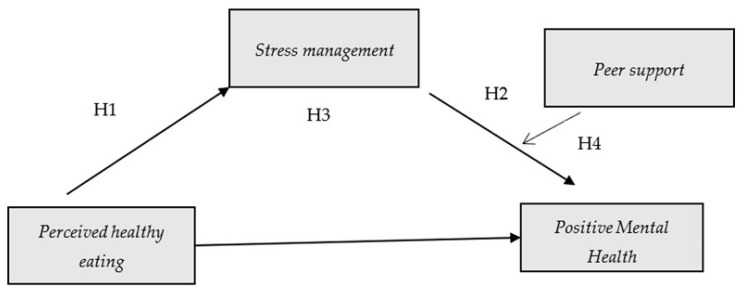
**Moderated Mediation Model.** This figure exhibits the relationship among perceived healthy eating, stress management and positive mental health (PMH). Stress management is hypothesized to mediate the relationship between perceived healthy eating and PMH, while peer support is expected to moderate de association between stress management and PMH.

By adopting a salutogenic perspective, our study advances this field by examining how perceived healthy eating operates not isolation, but through intrapersonal (i.e., stress management) and interpersonal (i.e., peer support) resources. This approach expands the literature from associations with psychopathology toward a holistic model of flourishing and provides novel insights with direct implications for school-based health promotion [31,32]. The purpose of this study is therefore to investigate these influencing mechanisms within a moderated mediation framework. Thus, we introduce the proposed moderated mediation model (Figure 1), whose implications will be examined in the Results and Discussion sections.

Consequently, and based on existing literature and the conceptual framework guiding this study, we formulated the following hypotheses:

**H1.**  *Perceived healthy eating will be positively associated with stress management in adolescents.*

**H2.**  *Stress management will be positively associated with PMH.*

**H3.**  *Stress management will mediate the relationship between perceived healthy eating and PMH: adolescents perceiving healthier eating habits will report better stress regulation which, in addition, will be associated with greater PMH.*

**H4.**  *Peer support will moderate the relationship between stress management and PMH: the higher the peer support, the stronger the positive influence of stress management on PMH.*

## 2. Materials and Methods

### 2.1. Participants and Setting

A cross-sectional study was conducted with a sample of 505 adolescents, aged 13–15, recruited from various urban school-based environments. The study protocol was approved by the Ethics Committee of the Príncipe de Asturias University Hospital (reference number OE 07/2023) on 1 February 2023. The Declaration of Helsinki guideline was followed during the study, and informed consent was obtained from parents and adolescents for their participation. The present study is part of a broader research project focused on PMH in adolescents and its associations with cognitive-emotional variables, lifestyle factors, and interpersonal relationships.

Eligibility criteria required participants to (a) be officially enrolled in the third year of compulsory secondary education (ESO) at the time of data collection and (b) demonstrate sufficient reading comprehension and digital skills to independently complete the online questionnaire. Students were excluded if they (a) were on medical leave or temporarily suspended during the study period or (b) had a diagnosed disability that could hinder their ability to understand the questionnaire items.

### 2.2. Data Collection

An ad hoc online questionnaire was developed for this study. The first part of the questionnaire included self-reported data on sociodemographic variables such as age, gender, nationality, family situation, educational support and grade repetition.

The second part of the questionnaire assessed PMH by using the Positive Mental Health Questionnaire (PMHQ), which is a self-administered questionnaire with 39 items, that was originally developed in Spanish by Lluch-Canut [33], and it has also been validated for the Portuguese population by Sequeira et al. [34]. The PMHQ contains 36 items loaded into six factors of the Multi-Model of Positive Mental Health—personal satisfaction (F1), prosocial attitude (F2), self-control (F3), autonomy (F4), problem solving and self-realization (F5), and interpersonal relationship skills (F6). This form has obtained a good global internal consistency of 0.92, with factors ranging from 0.60 to 0.82 and has shown adequate psychometric properties in adolescent populations (from 11 to 20 years) in previous studies [35].

Perceived healthy eating, stress management, and peer support were assessed using single-item self-report measures, designed to capture adolescents’ broad, subjective perceptions in a brief and accessible manner. The stress management and peer support were measured using questions rated on a 5-point Likert scale from 0 (“never”) to 4 (“very frequently”): ‘To what extent do you feel capable of controlling your distress?’, ‘In the last month, have you felt supported by your friends when you needed it?’. Perceived healthy eating was assessed with a single direct item asking adolescents to rate how healthy they considered their own eating. This measure was selected to capture perceived diet quality (self-rated diet), a psychological construct distinct from quantitative measures of intake. The question ‘To what extent do you consider your diet to be healthy?’ was asked and it was also evaluated on a 5-point Likert scale from ‘not at all’ to ‘very much’.

All these items were formulated based on relevant theoretical considerations. The choice was made due to strict time constraints in the school-based data collection context, where longer scales were not feasible without risking participant fatigue. These constructs were framed as global perceptions rather than multidimensional traits, which supports the appropriateness of single-item assessment [36,37,38,39,40,41]. Previous research has shown that single-item measures can yield acceptable levels of reliability and validity, particularly when assessing unidimensional constructs and in contexts requiring brief assessments, such as large-scale or time-constrained studies [42,43,44,45]. Research also shows that a single global measure of perceived diet quality can be a valid low-burden screener [37,39,40]. The same can be established for social support and related psychosocial constructs [36,38,41]. Moreover, students had received curricular instruction on healthy lifestyles, including nutrition, which likely provided a shared conceptual framework for interpreting the items. In addition, school-based nutrition education has been shown to be associated with improved knowledge and attitudes about healthy eating, favoring a clearer conceptualization of what constitutes “healthy eating” [46,47,48,49]. Accordingly, and in line with WHO guidance and recent reviews of healthy dietary patterns [50,51], a healthy diet can be broadly defined as “patterns higher in fruits, vegetables, whole grains, legumes, nuts and seeds, and lower in saturated/trans fats, added sugars and excess salt”.

Before beginning data collection, the study objectives and procedures were explained to the school administration, and the necessary authorizations were obtained. Information sheets and parental consent forms were distributed to the legal guardians of all eligible students. Only adolescents whose parents signed the informed consent form and who also gave their own consent were included, with both permissions being revocable at any time.

The online questionnaire was administered during regular classes, integrated into the school timetable, and supervised jointly by school staff and members of the research team. The survey, created using Google Forms, required participants to answer each question before moving on to the next, ensuring that all responses were complete. Consequently, it was not necessary to resort to imputation or deletion techniques to deal with missing data.

The self-administered questionnaire took approximately 35 min to complete. During administration, researchers were present to clarify questions and offer guidance. Data collection was conducted anonymously, with strict security measures in place to protect participant confidentiality. A pilot test was also conducted with a small group of adolescents to confirm the clarity and comprehensibility of the questions.

### 2.3. Statistical Analysis

Statistical analyses were performed using IBM SPSS Statistics 27.0 [52]. Descriptive statistics (mean, standard deviation, minimum, maximum, and frequencies) provided an overview of the data. The Kolmogorov–Smirnov test showed non-normal distributions for PMH (K-S = 0.053, *p* < 0.001), which is common in psychological research [53]. Despite this, parametric tests were used due to the large sample size (*n* = 505), as the Central Limit Theorem supports their robustness in such cases. Specifically, parametric tests remain reliable even when the assumption of normality is violated in large samples. Additionally, as has been pointed out, psychological variables frequently show non-normal distributions due to their intrinsic characteristics, yet parametric approaches are still considered suitable in these cases [54,55].

To identify covariates, *t*-tests and one-way ANOVAs were conducted for group comparisons. Pearson correlation coefficients were also used to identify possible covariates and examine the associations between variables in the proposed model. This analysis served as a foundational step for conducting the mediation analysis [56].

Mediation and moderated mediation analyses were conducted using the PROCESS macro for SPSS. Model 4 tested whether stress management mediated the link between perceived healthy eating and PMH. Model 14 assessed whether this mediation was moderated by peer support, conceptualized as a psychosocial resource. Indirect effects were evaluated using bootstrapping (5000–10,000 resamples), with significance determined by 95% confidence intervals excluding zero [57]. A *p*-value threshold of <0.05 was applied throughout.

To reduce potential confounding and bias, our statistical models included controls for gender, age, nationality, and family and educational variables.

## 3. Results

### 3.1. Sample Characteristics

Table 1 presents the socio-demographic and academic profile of the student sample. The study included 505 students with an average age of 13.62 years (SD = 0.70), and a nearly equal distribution of boys and girls (approximately 50% each). All participants were enrolled in schools located in a town northeast of Madrid, Spain. The majority were Spanish nationals (88.1%), with the most common foreign nationalities being Venezuelan (2.7%, *n* = 14), Romanian (2.4%, *n* = 12), and Colombian (1.6%, *n* = 8). Regarding parental nationality, around 70% of the parents were Spanish.

In terms of family structure, most students (69.7%, *n* = 352) lived in households with both parents, while 22.8% (*n* = 115) came from separated or divorced families. The remaining students belonged to single-parent, same-parent, or foster families, among other arrangements.

Academically, 29.5% of students (*n* = 149) had repeated a grade. Nearly half (43.8%, *n* = 221) received some form of educational support. The most common types were private tutoring (26.7%, *n* = 135), participation in diversification programs (7.5%, *n* = 38), and specific educational adaptations (2%, *n* = 10).

### 3.2. Descriptive Statistics and Correlations

Table 2 shows the descriptive statistics and correlations among the psychosocial variables examined. As can be seen, the mean value for the ability to manage stress is 2.10 (SD = 1.05), for the perception of healthy eating is 1.99 (SD = 0.99) and for peer support is 2.62 (SD = 1.15), all of them in a response range of 0 to 4. Scores on PMH can be considered high (Mean = 118.33; SD = 15.01). Regarding correlations, significant and positive correlations were observed between all variables of interest. The highest correlations were observed between PMH and stress management, perceived healthy eating, and peer support (all *p* < 0.001).

### 3.3. Preliminary Analyses: Covariates

The covariate analysis aimed to examine the possible relationship of the variables involved in the model (perceived healthy eating, stress management, peer support and PMH) with the sociodemographic variables considered. Sociodemographic factors previously highlighted in the literature as relevant to mental health—such as age, gender, educational support and family structure—have been included as potential covariates [58,59]. Table 3 shows the significant relationships found.

Age shows a statistically significant association with peer support (F = 7.67, *p* < 0.001). More precisely, 13-year-olds (M = 2.72, SD = 1.07) report higher levels of peer support compared to 15-year-olds (M = 2.11, SD = 1.33; *p* = 0.001). Similarly, 14-year-olds (M = 2.66, SD = 1.14) also differ significantly from the 15-year-old group (*p* = 0.004).

Gender differences were found to be statistically significant in both stress management (t = 7.128; *p* < 0.001) and PMH (t = 5.871; *p* < 0.001). Women reported lower scores in stress management (M = 1.79, SD = 0.96) compared to men (M = 2.42, SD = 1.04). Similarly, PMH scores were lower among women (M = 114.64, SD = 15.27) than men (M = 122.24, SD = 13.72).

Nationality (Spanish vs. non-Spanish) is significantly associated with both PMH (t = 3.639; *p* < 0.001) and peer support (t = 3.770; *p* < 0.001). Spanish participants reported higher PMH scores (M = 119.17, SD = 14.88) compared to non-Spanish participants (M = 111.62, SD = 15.43). Similarly, peer support was greater among Spanish adolescents (M = 2.70, SD = 1.12) than among their non-Spanish counterparts (M = 2.10, SD = 1.22). Differences were also observed in perceptions of healthy eating (t = 2.027, *p* = 0.043), with Spanish participants reporting healthier dietary perceptions (M = 2.02, SD = 0.97) than non-Spanish participants (M = 1.74, SD = 1.09).

Grade repetition is significantly related to both PMH (t = −2.311; *p* = 0.021) and peer support (t = −2.038; *p* = 0.042). Students who have repeated a grade tend to report lower PMH scores (M = 115.95, SD = 14.88) and lower levels of peer support (M = 2.46, SD = 1.27) compared to those who have not repeated (PMH: M = 119.32, SD = 14.98; peer support: M = 2.69, SD = 1.09).

Receiving additional educational support is significantly associated with lower levels of PMH (t = 3.311, *p* = 0.001). Students who receive such support report lower PMH scores (M = 115.97, SD = 15.08) compared to those who do not (M = 120.43, SD = 14.17). Differences are also evident in perceived healthy eating (t = 2.329, *p* = 0.020). Students receiving educational support report lower scores in their perception of healthy eating (M = 1.88, SD = 0.98) compared to those who do not receive such support (M = 2.09, SD = 0.97).

No significant relationships are observed between family structure and the variables involved in the model.

### 3.4. Inferential Analyses: Theoretical Models

#### 3.4.1. Simple Mediation Model (Perceived Healthy Eating, Stress Management and PMH)

Table 4 shows the results of the simple mediation analysis. According to the analysis of the possible covariates carried out in the previous section (see Table 3), age, gender, nationality, grade repetition and educational support should be included as covariates.

The proposed model identified stress management as a mediating variable in the relationship between perceived healthy eating and PMH. Findings indicated that perceived healthy eating was significantly associated with PMH (Total effect of X on Y: Effect = 3.009, SE = 0.658, t = 4.567, *p* < 0.001, 95% CI = 1.714 to 4.304). Additionally, a significant direct effect of perceived healthy eating on PMH was observed (Effect = 1.728, SE = 0.599, t = 2.884, *p* = 0.004, 95% CI = 0.551 to 2.906). The indirect effect of perceived healthy eating on PMH via stress management was also statistically significant (Effect = 1.280, SE = 0.343, 95% CI = 0.657 to 1.996). Both the partially and fully standardized indirect effects were significant as well (Partially standardized: Effect = 0.086, SE = 0.022, 95% CI = 0.045 to 0.132; Fully standardized: Effect = 0.084, SE = 0.022, 95% CI = 0.044 to 0.129).

**Figure 2:**
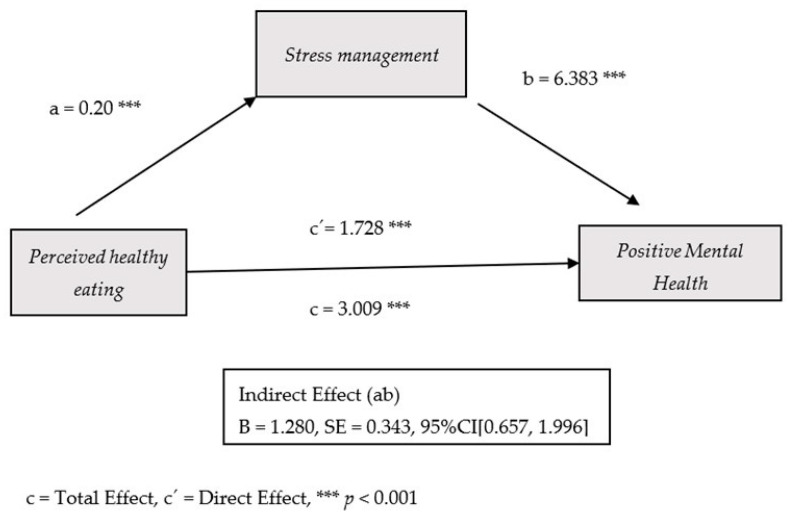
**Coefficients of the proposed simple mediation model.** The figure illustrates the significant indirect effect of perceived healthy eating on positive mental health (PMH) via stress management. Path *a* reflects the positive link between perceived healthy eating and stress management. Path *b* reflects the positive association between stress management and PMH. The direct effect of perceived healthy eating on PMH remains significant even after including the mediator.

The model accounts for 32% of the variance in PMH, as indicated by a significant overall fit (F = 30.58, *p* < 0.001). Specifically, perceived healthy eating shows a positive association with stress management (a = 0.200, SE = 0.046, t = 4.286, *p* < 0.001, 95% CI = 0.108 to 0.292). In turn, stress management was positively associated with PMH (B = 6.383, SE = 0.582, t = 10.958, *p* < 0.001, 95% CI = 5.238 to 7.528). Regarding covariates, gender significantly affects stress management (Effect = −0.622, SE = 0.091, t = −6.790, *p* < 0.001, 95% CI = −0.802 to −0.442), while nationality (Effect = −5.376, SE = 1.843, t = −2.916, *p* = 0.003, 95% CI = −8.998 to −1.753) and educational support (Effect = −2.440, SE = 1.178, t = −2.071, *p* = 0.038, 95% CI = −4.756 to −0.124) had a significant impact on PMH. Figure 2 illustrates the path coefficients among the variables included in the model.

#### 3.4.2. Moderated Mediation Model (Peer Support as a Moderating Variable)

The following analyses aimed to test a moderated-mediation model that included the simple mediation model, peer support as a moderated variable between stress management and PMH (Model 14). Table 5 shows the results of the moderate-mediation analysis.

As can be seen in Table 5, perceived healthy eating was positively associated with stress management (a = 0.200, SE = 0.046, t = 4.286, *p* < 0.001, 95% CI = 0.108, 0.292). In turn, stress management had a positive relationship with PMH (B = 8.719, SE = 1.341, t = 6.497, *p* < 0.001, 95% CI = 6.082, 11.356). Direct effects of perceived healthy eating on PMH are observed (c’ = 1.288, SE = 0.584, t = 2.205, *p* = 0.027, 95% CI = 0.140, 2.436).

**Figure 3:**
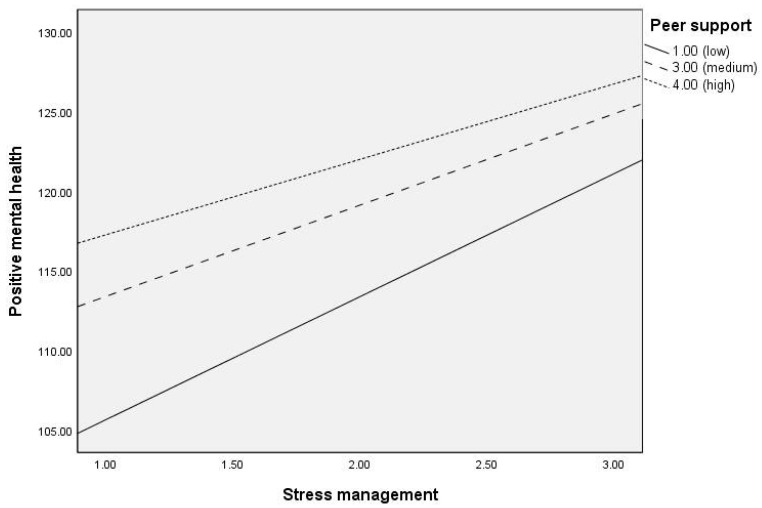
**Conditional effects of Peer Support on the relationship between Stress management and Positive mental health (PMH).** The figure indicates that peer support moderates the positive relationship between stress management and PMH. More precisely, this relationship is stronger under conditions of low peer support and weaker when peer support is high, implying that stress management plays a particularly important role in sustaining PMH when social support from friends is scarce.

Regarding the moderating variable, peer support had a direct positive association with PMH (Effect = 4.863, SE = 1.107, t = 4.391, *p* < 0.001, 95% CI = 2.687, 7.039). Similarly, a significant interaction effect of peer support on the relationship between stress management and PMH was observed (Effect = −0.995, SE = 0.464, t = −2.144, *p* = 0.032, 95% CI = −1.907, −0.083). Table 6 shows the conditional effects of stress management in PMH as a function of peer support. As can be seen, as scores on peer support become higher, the effects of stress management on PMH are minor (in all cases statistically significant). Figure 3 also shows these results.

In terms of covariates, significant associations were observed for gender in stress management (Effect = −0.622, SE = 0.091, t = −6.790, *p* < 0.001, 95% CI = −0.802, −0.442), and for gender (Effect = −2.944, SE = 1.171, t = −2.513, *p* = 0.012, 95% CI = −5.247, −0.642), nationality (Effect = −4.131, SE = 1.797, t = −2.298, *p* = 0.022, 95% CI = −7.664, −0.599) and educational support (Effect = −2.301, SE = 1.139, t = −2.018, *p* = 0.044, 95% CI = −4.540, −0.061) in PMH.

The proposed model contributed to the explanation of 36% of the variance of PMH (F = 29.28, *p* < 0.001).

In addition to the objectives and hypotheses raised in this manuscript, and given the correlational nature of this research and the bidirectional relationships established between the variables involved (see Table 2), alternative models were additionally tested regarding the relationship between the perception of healthy eating, stress management and PMH. Thus, one model posits the mediating role of perceived healthy eating (M) between stress management (X) and PMH (Y), while the other posits the mediating role of stress management (M) between PMH (X) and perceived healthy eating (Y). In both models, the moderating role of peer social support between M and Y is maintained. Both models were tested but did not yield sufficiently significant results. In the first model, stress management is significantly associated with perception of healthy eating (B = 0.19, *p* < 0.001), but perception of healthy eating was not significantly associated with PMH (B = 2.25, *p* = 0.09). Peer social support showed significant direct effects (*p* = 0.004), but no interaction effects (*p* = 0.926). In the second model, PMH was significantly associated with stress management (B = 0.034, *p* < 0.001), and showed a direct effect on perception of healthy eating (B = 0.012, *p* = 0.005); however, stress management is not significantly associated with perception of healthy eating (*p* = 0.204), and peer social support showed no significant direct (*p* = 0.135) or interaction effects (*p* = 0.711).

## 4. Discussion

The present study provides evidence for a moderated mediation model linking perceived healthy eating to PMH through stress management, with peer support acting as a significant moderator. These findings could suggest that adolescents who perceive themselves as eating healthier are more likely to regulate stress effectively, which, additionally, enhances their PMH. These associations remained significant even after controlling for gender, age, nationality and educational variables.

Evidence from different adolescent populations points to the association between nutrition and mental health indicators. A Spanish study showed that adolescents (aged between 12 and 16 years old) with low adherence to the Mediterranean diet reported lower satisfaction of basic psychological needs compared to those with medium or high adherence, regardless of their activity status [60]. On the other hand, a large-scale study in Korean adolescents found a decline in the consumption of healthy foods (e.g., breakfast, vegetables, fruit, milk) alongside rising obesity, with dietary habits linked to sleep quality, depression, and stress [61]. Similarly, research in Spanish university students showed that unhealthy eating patterns were highly prevalent, especially among women, and were also associated with greater anxiety, depression, stress, and sleep problems, particularly in relation to high intake of sweets and low dairy consumption [62]. These patterns reinforce the notion that inadequate dietary habits developed during adolescence may persist in adulthood, contributing to a sustained risk of psychological distress and impaired well-being over time.

It is also well known that psychological resources play a key role in explaining how behaviors, such as nutrition, impact on mental health. For example, a study conducted among college freshmen found that higher perceived stress was linked to increased consumption of sugary drinks, fast food, and salty snacks. Noticeably, the relationship between stress and sweet snack consumption was stronger among students who reported low stress management ability, highlighting the moderating role of emotional coping resources in the stress-nutritional link [63]. Similarly, our findings showed that adolescents who perceived their eating habits as healthy also presented better stress management, which in turn was related to better PMH. These patterns are particularly relevant during early adolescence, a developmental stage marked by ongoing maturation of emotional regulation, heightened sensitivity to rewards, and growing independence in lifestyle choices [64,65,66]. In this context, perceiving one’s diet as healthy may serve as a valuable psychological resource, helping adolescents cope with stress and maintain PMH.

Besides, while our study identified stress management as a key mediator between perceived healthy eating and PMH, other studies have pointed to related constructs, such as emotional self-regulation or self-concept. In this sense, a recent study among pre-adolescents found that healthier dietary habits (i.e., higher intake of fruits and vegetables) were associated with fewer emotional and behavioral problems, and that this relationship was significantly mediated by children’s self-concept [15]. That is, children who ate more nutritious foods reported a more positive self-concept, which, in addition, was linked to better emotional and behavioral outcomes. Likewise, Mancone et al. [16] demonstrated that nutrition education interventions fostering food literacy and self-regulation skills led to improvements in adolescents’ eating behaviors and overall health outcomes. It should be noted that although self-concept and stress regulation are conceptually distinct, both reflect the adolescent’s internal capacity to manage emotional challenges and may interact or reinforce each other promoting psychological resilience. Moreover, they could be understood as psychological antecedents of PMH given their close association with emotional well-being and adaptative functioning.

Interestingly, recent research highlights the multifaceted role of health-enhancing behaviors (HEB) in adolescent mental health, particularly beyond the traditional “big three” (i.e., nutrition, sleep, and physical activity). Bromley et al. [67] propose expanding this framework to include mindfulness and social connectedness, as these emerged as the strongest predictors of both psychological distress and well-being in early adolescence. While their main conclusions emphasize the primacy of social and emotional factors, a closer inspection of their multivariate models reveals that nutrition was independently associated with lower psychological distress, although it did not significantly predict well-being. In line with this, our findings indicate that adolescents who perceived their eating habits as healthy also reported higher stress management (β = 0.20, *p* < 0.001), which was associated with higher PMH (β = 8.71, *p* < 0.001). Notably, our results further showed that peer social support moderated this indirect pathway, such that the positive association between stress management and PMH is weaker in adolescents who report higher levels of peer support. Whereas Bromley and co-workers [67] identified social connectedness as a relevant direct predictor, our findings suggest that it may also affect the link of psychological regulation mechanisms, highlighting its potential as a contextual factor in mental health promotion.

In this vein, literature consistently has shown that peer support plays a crucial role in adolescents’ mental health, stress management, and health-related behaviors. Longitudinal findings indicate that high-quality peer relationships uniquely predict lower levels of depression during adolescence and even into adulthood [20]. Furthermore, peers strongly influence dietary choices and physical activity patterns, as adolescents frequently model their eating and exercise habits on those of their friends, which can either promote or hinder healthy lifestyles [68]. In line with the social buffering theory, peer support has been proposed to mitigate the negative effects of stress by reducing physiological reactivity and promoting better emotional regulation. However, empirical evidence in adolescents remains inconclusive. While some studies highlight the protective role of friendship in adverse contexts, others suggest that adolescents facing adversity may be more vulnerable to interpersonal stress and less able to mobilize social support effectively [69].

Within this scenario, our results provide new insights. As already mentioned, and contrary to our initial hypothesis (H4) which anticipated that greater peer support would enhance the relationship of stress management with PMH, our findings suggest that when peer support is low, stress management emerges as a key strategy for maintaining PMH. In contrast, when peer support is high, the influence of stress management on PMH becomes less pronounced. This pattern indicates that peer support may function as a relatively independent protective factor [70,71]. In other words, individuals with high levels of peer support may rely on these interpersonal resources rather than on individual stress-regulation strategies. Importantly, as shown in Table 5, peer support was positively and significantly associated with PMH (B = 4.863, *p* < 0.001).

This moderation effect could be understood by considering the developmental stage of the participants (i.e., early adolescence, 13–15 years). During this period, adolescents increasingly shift reliance from family to peers for emotional support and social regulation [66,72]. Adolescents with high peer support may perceive less need to employ individual stress management strategies, relying instead on interpersonal resources. Conversely, when peer support is low, adolescents must depend more on internal coping skills to maintain PMH. From a theoretical perspective, these findings align with Hobfoll’s Conservation of Resources theory [73], which posits that individuals seek to acquire, maintain, and protect valued resources, and that resources can serve compensatory roles (i.e., social support can act as a compensatory resource that reduces the reliance on intrapersonal coping strategies). These interpretations are also consistent with research on adolescent coping and resilience. Early adolescents are still consolidating individual stress regulation skills, and high social support may lead to a temporary recourse on peers rather than internal strategies [74,75]. The interaction observed highlights the dynamic interplay between individual and social resources in promoting mental health: both are beneficial, but their relative importance may vary depending on context and developmental stage. Moreover, from a perspective of autonomy and social learning, adolescents experiment with different sources of support, and high levels of peer support may reduce motivation to apply personal coping strategies because the peer network functions as an emotional buffer. This can be conceptualized through the social self-efficacy model: when adolescents trust the capacity of their peers to provide support, the relative contribution of their individual self-efficacy in stress management to overall well-being may be diminished, as social resources provide alternative or complementary protection against stress [76,77]. Integrating this perspective with the Conservation of Resources framework and developmental considerations may constitute a comprehensive explanation of why peer support might attenuate the influence of individual stress management on PMH in early adolescence.

Overall, these findings highlight the dynamic interplay between individual coping strategies and social resources in promoting adolescent PMH, aligning with broader theoretical models of psychological well-being. For instance, Seligman’s PERMA model (positive emotion, engagement, relationships, meaning, and accomplishment) [78,79] provides a multidimensional framework for understanding how healthy behaviors such as regular physical activity, balanced nutrition, and adequate sleep contribute to youth mental wellness [4]. Recognizing developmental differences, adaptations of PERMA for adolescents, such as the EPOCH model, have emphasized five positive psychological traits particularly relevant for youth: engagement, perseverance, optimism, connectedness and happiness [80]. This version retains the core idea that well-being is multifaceted, while translating it into developmentally appropriate constructs for younger populations. In our study, perceived improvements in healthy eating and better stress management may foster not only emotional balance, but also a greater sense of control, meaning, and self-efficacy, dimensions that could be closely related to engagement, perseverance and optimism. Additionally, the moderating role of peer support could be interpreted in relation to the relational cornerstone or connectedness dimension of PERMA and EPOCH. Our findings resonate with this view, while also reinforcing the dynamic interplay between social and individual resources, suggesting that psychological strengths may mediate the influence of lifestyle factors on PMH outcomes in adolescents.

However, it is important to note that these frameworks primarily describe what flourishing looks like, rather than how health-promoting resources enable adolescents to thrive. In this regard, the salutogenic model offers a complementary perspective by focusing on the processes and resources that allow individuals to maintain well-being despite stressors [25,26,27,28]. Anchored in this model, our study examines perceived healthy eating not simply as a healthy behavior, but as a psychological and social resource that operates through stress management and is shaped by peer support. This process-oriented approach distinguishes PMH from the mere absence of psychopathology [31,32] and addresses a critical gap in the literature: while nutrition–mental health associations are well documented, few studies investigate perceived nutrition within a salutogenic framework or explain its indirect pathways to flourishing in adolescence. Our findings thus advance beyond descriptive associations by integrating lifestyle perceptions, coping skills, and social connectedness into a coherent explanatory model that suggests potential mechanisms underlying PMH.

Building on this, several multicomponent programs have demonstrated the potential of integrating physical, emotional, and social dimensions to improve adolescent well-being. For instance, the T-COPE Healthy TEEN program, a school-based initiative combining nutrition education, physical activity promotion, and stress management, evidenced improvements in adolescents’ fruit and vegetable intake, water consumption, physical activity, and anxiety symptoms, along with gains in health-related knowledge. Although no significant changes were observed in Body Mass Index or depression levels compared to controls, the program’s positive impact on daily behaviors and emotional regulation supports the idea that addressing both physical and psychological dimensions can enhance youth well-being [81]. In light of our findings, such programs may be particularly effective when they integrate both individual behaviors and social-contextual factors. Likewise, as highlighted by Subramanyam et al. [82], promoting mental well-being in children and adolescents requires a comprehensive perspective that includes not only clinical or individual-level interventions, but also school-based initiatives, family dynamics, and broader socio-environmental conditions. Elements such as nutrition, physical activity, inclusive education, and supportive social settings should be considered essential components of any strategy aimed at fostering long-term psychological well-being in this stage of life. Similarly, recent research highlights the importance of a multidisciplinary and context-sensitive approach to adolescent mental health. For example, need-based nursing interventions, specifically those addressing nutrition, injury prevention, and engagement in meaningful activities, could be highly relevant for children and adolescents with mental health disorders [83]. Attention should also be paid to the quality of adolescents’ social relationships, both in person and online, as there is evidence that social media use has a significant impact on diet [84,85]. The challenge lies in the fact that health professionals have the opportunity to use social media to promote healthy eating among adolescents.

### Limitations and Future Directions

This study presents several limitations that warrant consideration. Although our moderated mediation model is grounded in theory, the findings should be interpreted as indicative of associations rather than casual effects. First, its cross-sectional design limits the ability to draw causal inferences or track developmental changes. Given that adolescence is a dynamic and evolving period, future research would benefit from longitudinal designs to examine how perceived nutrition, stress control and peer support interact and evolve over time in relation to PMH outcomes. Furthermore, intervention studies are needed to evaluate the effectiveness of programs that promote healthy lifestyles, enhancing adolescents’ ability to manage stress and strengthen the supportive role of both peers and family relationships as protective factors for mental well-being.

Second, the study relied exclusively on self-report measures, which can introduce biases such as social desirability or memory-related inaccuracies. The inclusion of multi-informant approaches, such as parent, teacher, or peer reports, as well as observational methods, could enhance data validity and provide a more comprehensive picture of adolescent psychological functioning.

Third, while single-item measures are increasingly used in large-scale adolescent health surveys and within school contexts due to feasibility and reduced respondent burden, they inevitably represent a significant methodological limitation. Although the instruments assessing the constructs considered in our study (i.e., perceived healthy eating, stress management and social support) were developed based on theoretical models, they were created ad hoc for this study rather than adapted from previously validated multi-item scales. While as already-mentioned this approach allowed for brevity and feasibility within the school setting, it may have limited both reliability and construct validity. Therefore, future research should consider incorporating standardized, validated tools to strengthen measurement precision and comparability. Nevertheless, several procedural and contextual factors may have mitigated potential bias. Specifically, participating students had previously received school-based nutrition education, which likely provided a shared curricular framework for interpreting the items, and the analysis statistically controlled for demographic and educational covariates (i.e., gender, age, nationality and educational variables).

Finally, the sample characteristics limit the generalizability of the findings. The study focused on adolescents aged 13–15 from urban schools, which may not reflect the experiences of youth from other age ranges, rural areas, or different sociocultural contexts. Replication in more diverse and heterogeneous populations would help determine the broader applicability of the results.

Conscious of these limitations, supplementary sensitivity and dose–response analyses were conducted to further support the robustness of the findings. Sensitivity checks—including exclusion of covariates, non-parametric comparisons, subgroup testing, and variation in bootstrap resampling—confirmed the stability of the results. Additionally, a dose–response analysis using ordinal levels of healthy eating (low, medium, high) revealed a positive gradient in its association with PMH, reinforcing the validity and interpretability of the proposed model.

Beyond research implications, these findings also highlight the need for action at the practical and policy levels. For practitioners and policymakers, this underscores the importance of designing multisectoral, developmentally appropriate interventions that not only treat mental health problems but also actively promote resilience and psychological flourishing from early-life stages. However, given the cross-sectional design and the use of single-item measures, these implications should be viewed as preliminary and hypothesis-generating rather than prescriptive. They intend to inform future research and intervention design by suggesting that programs integrating both individual (e.g., stress management, perceived healthy eating) and social-contextual factors (e.g., peer support) could be promising avenues for adolescent well-being promotion. Nurses, as key members of care teams, are well positioned to implement holistic, strength-based interventions that extend beyond hospital settings into primary care and community mental health systems, contributing to sustainable and developmentally appropriate mental health promotion.

## 5. Conclusions

This study provides preliminary empirical evidence for a moderated-mediation model in which adolescents’ perceived healthy eating is associated with higher levels of PMH through the mediating role of stress management. Importantly, this indirect pathway appears to be moderated by peer social support, suggesting that the positive effect of stress management on PMH may be attenuated among adolescents who report higher levels of peer support. These associations remain significant after controlling for age, gender, nationality and educational variables, underscoring the relevance of psychosocial factors in the promotion of adolescent mental health.

By identifying stress management as a key psychological mechanism linking dietary perceptions to PMH, this research adds to the growing literature on modifiable lifestyle behaviors and their relationship with youth well-being. Moreover, the results are consistent with existing theoretical frameworks, such the PERMA and EPOCH models, which highlight the role of psychological resources like emotional regulation, self-concept, and connectedness in fostering resilience and personal growth.

In spite of this, given the cross-sectional design and reliance on self-report, ad hoc single item measures, the conclusions should be interpreted with caution. The present study should be viewed as an exploratory step that highlights potentially meaningful pathways between lifestyle perceptions and PMH during adolescence. Future longitudinal and cross-cultural research using validated multi-item measures is needed to confirm and extend these findings.

## Figures and Tables

**Table 1:** Socio-demographic characteristics of participants.

Characteristics	*n*	%	Mean	SD	Max	Min
Age			13.62	0.70	13	15
13 years old	257	50.9				
14 years old	184	36.4				
15 years old	64	12.7				
Nationality (Spanish)	446	88.1				
Gender						
Women	260	51.5				
Men	245	48.5				
Family situation						
Parents living together	352	69.7				
Divorced/separated parents	115	22.8				
Other family models	38	7.5				
Grade repetition (yes)	149	29.5				
Educational support (yes)	221	43.8				

SD: Standard Deviation; Max: Maximum; Min: Minimum.

**Table 2:** Descriptive statistics and correlations between variables.

	Mean	SD	Range	Asym	Kurt	2	3	4
1. Perceived healthy eating	1.99	0.99	0–4	−0.03	−0.52	0.21 **	0.18 **	0.27 **
2. Stress management	2.10	1.05	0–4	−0.112	−0.521		0.09 *	0.41 **
3. Peer support	2.62	1.15	0–4	−0.68	−0.27			0.27 **
4. Positive Mental Health	118.33	15.01	65–155	−0.41	0.027			

** *p* < 0.01; * *p* < 0.05; SD: Standard Deviation; Asym: Asymmetry; Kurt: Kurtosis.

**Table 3:** Analysis of possible covariates (only statistically significant results are shown).

	Perceived Healthy Eating	Stress Management	Peer Support	Positive Mental Health
Age			Yes (*p* < 0.001)	
Gender		Yes (*p* < 0.001)		Yes (*p* < 0.001)
Nationality	Yes (*p* = 0.043)		Yes (*p* < 0.001)	Yes (*p* < 0.001)
School retention			Yes (*p* = 0.042)	Yes (*p* = 0.021)
Receives educational support	Yes (*p* = 0.020)			Yes (*p* < 0.001)

**Table 4:** Simple mediation model: Effects of perceived healthy eating on positive mental health through stress management (Model 4).

DV: Stress Management	R^2^	F	*p*	Beta	SE	t	*p*
Model summary	0.14	12.12	<0.001				
IV: Perceived healthy eating				0.200	0.046	4.286	<0.001
Age (covariate)				−0.039	0.092	−0.424	0.671
Gender (covariate)				−0.622	0.091	−6.790	<0.001
Nationality (covariate)				0.122	0.146	0.834	0.404
Educational support (covariate)				−0.033	0.093	−0.353	0.723
Grade repetition (covariate)				0.118	0.144	0.821	0.411
**DV: Positive mental health**							
Model summary	0.32	30.58	<0.001				
IV: Perceived healthy eating				1.728	0.599	2.884	0.004
M: Stress management				6.383	0.582	10.958	<0.001
Age (covariate)				0.823	1.167	0.705	0.480
Gender (covariate)				−2.343	1.206	−1.942	0.052
Nationality (covariate)				−5.376	1.843	−2.916	0.003
Educational support (covariate)				−2.440	1.178	−2.071	0.038
Grade repetition (covariate)				2.525	1.815	1.391	0.164

DV: Dependent variable; IV: Independent variable; M: Mediator.

**Table 5:** Moderated—Mediation model: Perceived healthy eating—Stress management—Positive mental health using Peer Support (Model 14).

DV: Stress Management	R^2^	F	*p*	Beta	SE	t	*p*
Model summary	0.14	12.126	<0.001				
IV: Perceived healthy eating				0.200	0.046	4.286	<0.001
Age (covariate)				−0.039	0.092	−0.424	0.671
Gender (covariate)				−0.622	0.091	−6.791	<0.001
Nationality (covariate)				0.122	0.146	0.834	0.404
Educational support (covariate)				−0.033	0.093	−0.353	0.723
Grade repetition (covariate)				0.118	0.144	0.821	0.411
**DV: Positive mental health**							
Model summary	0.36	29.285	<0.001				
IV: Perceived healthy eating				1.288	0.584	2.205	0.027
M: Stress management				8.719	1.341	6.497	<0.001
Mo: Peer Support				4.863	1.107	4.391	<0.001
Interaction M * Mo				−0.995	0.464	−2.114	0.032
Age (covariate)				1.443	1.133	1.273	0.203
Gender (covariate)				−2.944	1.171	−2.513	0.012
Nationality (covariate)				−4.131	1.797	−2.298	0.022
Educational support (covariate)				−2.301	1.139	−2.018	0.044
Grade repetition (covariate)				3.039	1.757	1.729	0.084

DV: Dependent variable; IV: Independent variable; M: Mediator; Mo: Moderator; *: Interaction between the Mediator and the Moderator.

**Table 6:** Conditional effects of Peer support on the relationship between Stress management and Positive mental health.

Peer Support	Effect	SE	t	*p*	LLCI	ULCI
1 (16th percentile)	7.723	0.941	8.203	<0.001	5.873	9.573
3 (50th percentile)	5.732	0.592	9.682	<0.001	4.568	6.895
4 (84th percentile)	4.736	0.853	5.547	<0.001	3.058	6.414

SE: Standard error; LLCI: Lower-level confidence interval; ULCI: Upper-level confidence interval.

## Data Availability

The data supporting the results presented can be requested from the corresponding author if necessary.

## Abbreviations

The following abbreviations are used in this manuscript:

PMH	Positive Mental Health
